# Enhancing Hydrophilicity of Thick Electrodes for High Energy Density Aqueous Batteries

**DOI:** 10.1007/s40820-023-01072-y

**Published:** 2023-04-10

**Authors:** Jungeun Lee, Hyeonsoo Lee, Cheol Bak, Youngsun Hong, Daeha Joung, Jeong Beom Ko, Yong Min Lee, Chanhoon Kim

**Affiliations:** 1 Sustainable Technology and Wellness R&D Group, Korea Institute of Industrial Technology (KITECH), 102 Jejudaehak-Ro, Jeju-Si, Jeju-do 63243 Republic of Korea; 2 Department of Energy Science and Engineering, Daegu Gyeongbuk Institute of Science and Technology (DGIST), Daegu, 42988 Republic of Korea; 3 Department of Physics, Virginia Commonwealth University, Richmond, VA 23284 USA

**Keywords:** Thick electrodes, Hydrophilic binder, Sulfonation, Aqueous zinc-ion batteries, High areal capacity

## Abstract

Thick electrodes can substantially enhance the overall energy density of batteries. However, insufficient wettability of aqueous electrolytes toward electrodes with conventional hydrophobic binders severely limits utilization of active materials with increasing the thickness of electrodes for aqueous batteries, resulting in battery performance deterioration with a reduced capacity. Here, we demonstrate that controlling the hydrophilicity of the thicker electrodes is critical to enhancing the overall energy density of batteries. Hydrophilic binders are synthesized via a simple sulfonation process of conventional polyvinylidene fluoride binders, considering physicochemical properties such as mechanical properties and adhesion. The introduction of abundant sulfonate groups of binders (i) allows fast and sufficient electrolyte wetting, and (ii) improves ionic conduction in thick electrodes, enabling a significant increase in reversible capacities under various current densities. Further, the sulfonated binder effectively inhibits the dissolution of cathode materials in reactive aqueous electrolytes. Overall, our findings significantly enhance the energy density and contribute to the development of practical zinc-ion batteries.

**Supplementary Information:**

The online version contains supplementary material available at 10.1007/s40820-023-01072-y.

## Highlights

- Sulfonated polyvinylidene fluoride (S-PVdF) binders with improved hydrophilicity were synthesized via a simple sulfonation process of conventional hydrophobic PVdF binders.
- The abundant sulfonate groups of S-PVdF binders significantly improved ionic conduction in thick electrodes (~ 6 mg cm^−2^), enabling improved reversible capacities under various current densities.
- The S-PVdF binders effectively suppressed cathode dissolution, resulting in enhanced capacity retention at higher temperature operations (45 °C).

**Supplementary Information:**

The online version contains supplementary material available at 10.1007/s40820-023-01072-y.

## Graphical abstract

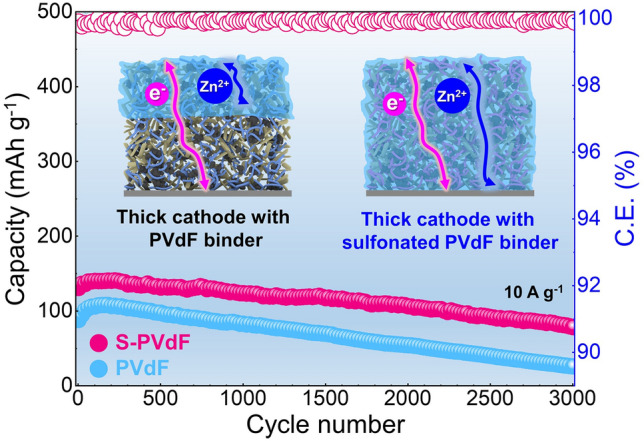

## Introduction

Aqueous zinc-ion batteries (ZIBs) based on Zn^2+^ intercalation chemistry have gained much attention owing to their vast advantages of zinc metal anodes, such as high gravimetric and volumetric energy densities (820 mAh g^−1^ and 5,855 mAh cm^−3^), relatively low redox potential (− 0.76 V vs. standard hydrogen electrode), abundance in nature, and low cost [1–9]. Moreover, aqueous ZIBs provide unparalleled safety, which is currently one of the biggest challenges of non-aqueous lithium-ion batteries (LIBs) [10]. Studies have been conducted to stabilize metallic zinc anodes (inhibition of zinc dendrites, corrosion, passivation, etc.) and explore new cathode materials for safer batteries [11, 12]. In particular, significant efforts have been made to develop nanoscale cathode materials such as manganese oxides, Prussian blue analogues, and vanadium-based materials with better structural integrity and short transportation path for Zn^2+^ migration [13, 14]. However, these nanoscale cathode materials have relatively larger surface areas than the same volume of bulk active particles, requiring a large amount of binders and conductive agents for adhesion and electrical connection inside the electrodes.

**Fig. 1:**
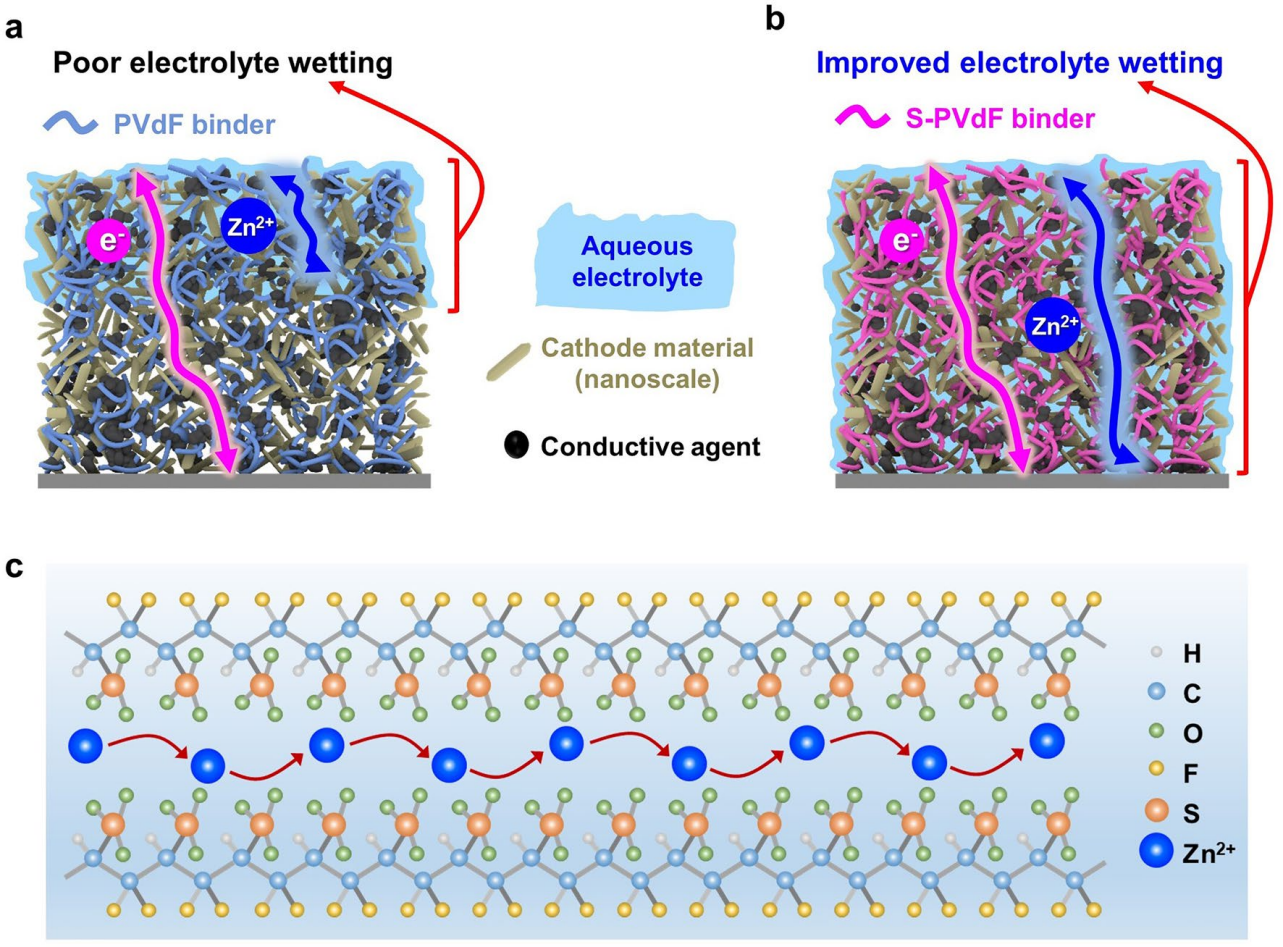
**a** Schematic illustrations show the transport limitation of Zn^2+^ in a thick electrode with PVdF binders. **b** Schematic illustrations show the facile transport of Zn^2+^ in a thick electrode with S-PVdF binders. **c** Zn^2+^ ion conduction via sulfonate groups in S-PVdF

In most current aqueous ZIB research, polyvinylidene fluoride (PVdF), the most conventional binder for LIBs, is still used despite its hydrophobic property, which leads to poor electrode wetting behavior, especially in aqueous electrolytes. Specifically, this occurrence will be aggravated by increasing the electrode thickness for higher areal capacity (> 2 mAh cm^−2^) because the constituent materials of electrodes for aqueous ZIBs are mixed with such nanoscale cathode materials and conductive agents, providing a long, tortuous pathway for bulk electrolyte percolation [15, 16]. The effective ionic diffusion in electrolytes can be expressed as:

1 $$D_{\text{eff}} = \frac{\varepsilon }{\tau }D_{0}$$

where* ε* is the porosity, *τ* is the tortuosity, and *D*_*0*_ is the diffusion coefficient of Zn^2+^ in electrolytes. According to the formula, *D*_eff_ is inversely proportional to the tortuosity of electrodes. Thus, the highly tortuous network of the thick electrode limits the infiltration of electrolytes and considerably increases the ion transfer pathway. As a result, active materials near the top of the thick electrode are more actively charged and readily discharged than those in the vicinity of the current collector, leading to insufficient utilization of active materials and reduced storage capabilities (Fig. 1a) [17]. Although a calendering process is essential for thick electrodes to increase the energy density of batteries, increasing calendering degree adversely influences the electrolyte wetting rate and the tortuosity. That is why the calendering process has been typically avoided in aqueous ZIB systems. Therefore, improving the wettability of the thick electrode and electrolyte permeability is critical to enhancing the utilization of nanoscale cathode materials and increasing energy density for aqueous ZIBs [18].

Within this subfield of thick electrodes, the role of the binders for aqueous ZIBs with nanoscale cathode materials is critical, as the binders significantly influence the hydrophilicity of electrodes. However, the performance of aqueous ZIBs with thick electrodes has been substantially limited by the hydrophobic PVdF binder borrowed from the conventional LIB system. We herein report sulfonated PVdF (S-PVdF) binders, which significantly increase the electrolyte wettability of thick electrodes for aqueous electrolytes. The S-PVdF binders are synthesized via a straightforward conventional sulfonation process with an optimizing degree of sulfonation considering physicochemical properties such as mechanical properties and adhesion. Indeed, abundant sulfonate groups in binders significantly increase electrolyte wettability, resulting in improved ionic conduction in thick electrodes even after the calendering process. More importantly, aqueous ZIBs with thick electrodes show highly improved reversible capacities under high current densities. Furthermore, the sulfonated PVdF binders effectively suppressed cathode dissolution, resulting in enhanced capacity retention at higher temperature operations. The devices demonstrated in this work serve as a proof of concept to provide feasible hydrophilic binders to further improve the energy density of aqueous ZIBs systems without changing the fundamental battery chemistries.

## Experimental

### Synthesis of S-PVdF Binders

All commercially available reagents and solvents were purchased from Merck and used as received without further purification. Briefly, 2 g of PVdF (average M_w_ ~ 1,100,000) powder was taken in a 50 mL vial, and chlorosulfonic acid (10 mL) was added slowly. The mixture was then heated at 80 °C for 0.5 h under constant stirring. The sulfonation degrees of S-PVdF was adjusted by the heating time from 0.5 to 4 h. Next, the mixture was precipitated in 1,2-dichloroethane and washed again with deionized water (DIW) until the pH reached 7. Subsequently, the mixture was put into a vacuum oven for drying at 60 °C for 24 h.

### Material Characterization

Surface and cross-sectional morphologies of electrode samples were characterized with a field-emission scanning electron microscopy (FE-SEM, MIRA3, TESCAN) operating at 10 kV and a Fourier transform infrared spectrometer (FT-IR, AlphaII, Bruker). To confirm the sulfonation of polymeric binders, proton nuclear magnetic resonance (^1^H NMR) spectra of binders were recorded at 298 K with a 600 MHz Agilent NMR spectrometer using deuterated N,N-dimethylformamide (DMF) as the solvent. All spectra were recorded in ppm units with tetramethylsilane (TMS) as an internal standard in the deuterated solvents. Surface chemical analysis was carried out using an X-ray photoelectron spectrometer system (XPS, AXIS SUPRA, Kratos Analytical) with the Al Kα X-ray source operating at 12 kV at the chamber pressure below 1 × 10^–8^ Torr and the spot size of 400 μm. Charge referencing was done with the carbon (C 1* s*) peak position of 284.8 eV. The wettability of electrodes was measured by a contact angle measurement using a 10 µL drop of electrolyte (2 M ZnSO_4_). The 3D surface roughness maps of cathodes with different binders were also investigated using a non-contact 3D optical surface profiler (NV-2400, Nanosystem Co. Ltd.). A surface and interfacial cutting analysis system (SAICAS, SAICAS-DN, Daipla Wintes, Japan) was used to measure the adhesive strength within the electrodes with different binders. The bulk adhesion within electrodes was repeatedly measured at every 10 μm depth from the surface with a boron nitride blade (width: 1 mm, rake angle: 20°, and clearance angle: 10°). The blade moved in the horizontal and vertical directions at 2.0 and 0.2 μm s^−1^, respectively. In addition, the interfacial adhesion strength at the electrode/current collector was measured under the cutting mode of 0.5 N and the peeling mode of 0.2 N. During the cutting mode, the blade approaches the interface with 0.5 N. After the blade contacts the interface, the blade peels the electrode with peeling mode, 0.2 N. To investigate the crystallinity of NH_4_V_4_O_10_, X-ray diffraction (XRD, Empyrean, Malvern Pan-alytical) with Cu Kα radiation (*λ* = 1.54056 Å) between 5° and 70° was used. Inductively coupled plasma optical emission spectroscopy (ICP-OES) analysis was carried out using an AVIO 550 (PerkinElmer).

### Cathode Preparation

A typical hydrothermal synthesis prepared the NH_4_V_4_O_10_ as the cathode active material. In brief, 3.5 g NH_4_VO_3_ was added to 360 mL of DIW and magnetically stirred at 70 °C until a light yellow solution was obtained. Then, 5.7 g of H_2_C_2_O_4_·2H_2_O was slowly added into the solution under magnetically stirring. Subsequently, the mixture was stirred for 6 h, transferred to a Teflon-lined autoclave, and heated at 140 °C for 48 h. After cooling and centrifugation, the product was rinsed with DIW several times. Finally, NH_4_V_4_O_10_ was obtained after drying in a convection oven at 80 °C for 12 h. Next, the NH_4_V_4_O_10_ was cast on stainless (SUS) foil and composed of three components: active material, conductive materials, and binders in the weight ratio of 7:2:1. Denka black was selected as the conductive material. Slurries containing the three components in N-methyl-2-pyrrolidone (NMP) were cast onto SUS foil (20 µm thick) via the doctor blading method, followed by a drying step at 80 °C for 12 h under vacuum. The active mass loadings for the cathode materials were ~ 2 mg cm^−2^ (electrodes with PVdF binders) and ~ 6 mg cm^−2^ (electrodes with S-PVdF and PVdF binders).

### Electrochemical Measurements

CR-2032 coin-type cells were assembled in ambient air by sandwiching glass fiber as a separator (Whatman) soaked with electrolyte (2 M ZnSO_4_ aqueous electrolyte) between the cathodes and Zn anodes. All galvanostatic measurements were tested using a WBCS-3000 battery cycler (Wonatech) in the potential window of 0.3–1.6 V at 25 °C. Cyclic voltammetry was also carried out using a ZIVE MP2A (Wonatech) at the same potential window at 25 °C. The electrochemical impedance spectroscopy (EIS) for evaluating ionic conductivity was obtained in the frequency range of 10^–2^–10^6^ Hz (at an AC voltage of 1 mV amplitude for ionic conductivity) using a potentiostat (ZIVE MP2A, Wonatech) at 25 °C.

To measure the ionic conductivities of binders, PVdF and S-PVdF films were prepared. The binders were dissolved in NMP solvent and magnetically stirred at 60 °C for 12 h. Then, the binder solutions were cast onto Al foil (20 µm thick) via the doctor blading method, followed by a drying step at 80 °C for 12 h under vacuum. The binder film-coated Al foils were immersed in 0.1 M NaOH solution by turning binder coated side down. Next, the detached binder films were soaked in the electrolyte (2 M ZnSO_4_ aqueous solution) for 24 h. The electrolyte-swelled binder films were sandwiched between stainless steel blocking electrodes in electrolyte and obtained a typical Nyquist plot in the frequency range from 1 Hz to 1 MHz. Then, the ionic conductivity was calculated from Eq. 2 [1]:

2 $$\sigma { } = \frac{l}{R \cdot A}$$

where *l* is the thickness of the hydrogel-based protective layer (20 μm), *A* is the contact area (1.13 cm^−2^), and *R* represents the resistance according to the EIS measurement at 25 °C.

## Results and Discussion

### Characterization of S-PVdF

Sulfonation is an industrial process used to manufacture a wide range of chemical products. It is a simple chemical reaction where sulfonate groups (–SO_3_H) are introduced on the hydrophobic polymer chains of PVdF, as shown in Fig. S1. This attachment allows the hydrophobic macromolecular chains of PVdF to align into the hydrophilic sulfonate group domains, improving its hydrophilicity (Fig. 1b). Furthermore, the sulfonate groups can perform as active sites to modulate ion transfer in PVdF due to its affinity with Zn^2+^ ions (Fig. 1c) [19]. The FTIR spectra of the sulfonated PVdF (S-PVdF) are shown in Fig. S2. The predominant peaks in the region of the 1035 and 1277 cm^−1^ (symmetric and asymmetric stretching of O=S=O), and 1012 cm^−1^ (S=O stretching) are for sulfonate groups [20, 21]. The ^1^H NMR spectroscopy was also used to confirm the sulfonation of PVdF, as seen in Fig. S3. Two characteristic peaks in the region of the 3.0–3.1 and 2.3–2.4 ppm are for head-to-tail and head-to-head bonding arrangements of semi-crystalline PVDF (H_A,A′_), respectively [22]. The peak shifts to lower ppm in the range of 6.2–6.5 ppm are attributed to the hydrogen attached to the sulfonated carbon (H_E_). The sulfonation degree (*D*_s_) of S-PVdF can be calculated by the following equations [23]:

3 $$\frac{n}{{{2} - {2}n}}{ = }\frac{{A_{{{\text{H}}_{{\text{E}}} }} }}{{\sum A_{{{\text{H}}_{{{\text{A,\,A}}^{\prime}}} }} }} \left( {0 \le n \le 1} \right)$$

4 $${D}_{\mathrm{S}}=n\times 100\%$$

where $A_{{{\text{H}}_{{\text{E}}} }}$ is the peak area of the H_E_ signal and $A_{{{\text{H}}_{{{\text{A, A}}^{\prime}}} }}$ is the integrated peak area of the signals corresponding to the other aliphatic hydrogens, and *n* is the number of H_E_ per repeat unit. The *D*_s_ of the S-PVdF was calculated to be 8%.

In general, with a strong binding affinity towards electrode components, the moderate ionic conductivity of binders is essential for shuttling ions between the electrolyte and active materials, which is induced by binder wetting in liquid electrolytes [24]. The non-sulfonated hydrophobic PVdF shows poor wetting behavior in aqueous electrolytes, exhibiting low ionic conductivity of 7.48 × 10^−5^ S cm^−2^ (Fig. S4). By contrast, the S-PVdF showed approximately onefold improvement in ionic conductivity (7.66 × 10^−4^ S cm^−2^) over the non-sulfonated PVdF. We have further confirmed that the sulfonate groups in S-PVdF binders can provide abundant coordination sites with Zn^2+^ by Fourier transformation infrared spectrum (FTIR) and X-ray photoelectron spectroscopy (XPS), as shown in Figs. S5 and S6. This observation is particularly beneficial to relieve serious concentration polarization of thicker electrodes, which is attributed to limited ion transport [25]. Furthermore, binders with high ionic conductivity can substantially improve the electrochemical performance of electrodes, especially for rate performance [26].

### Properties of Thick Electrodes with S-PVdF Binders

**Fig. 2:**
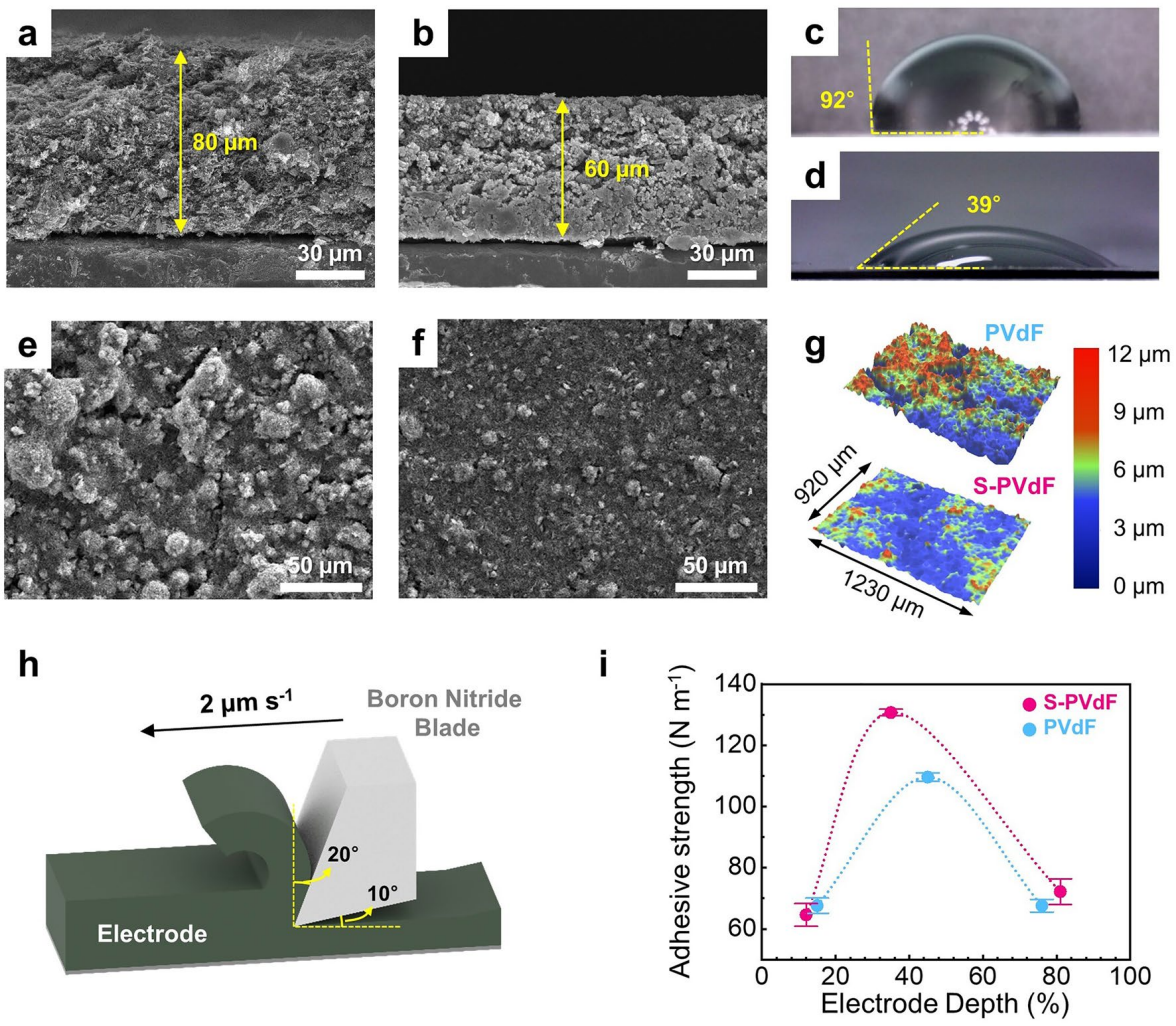
Cross-sectional SEM images of cathodes with S-PVdF binders **a** before and **b** after the calendering process. Contact angles of cathodes with **c** PVdF and **d** S-PVdF binders with 2 M ZnSO_4_ electrolyte. Top-view SEM images of cathodes with **e** PVdF and **f** S-PVdF binders. **g** 3D surface roughness maps of cathodes with different binders. **h** The schematic illustration of the SAICAS experiment. **i** The adhesive strength of the cathodes with S-PVdF and PVdF binders at various electrode depths

Thick electrode designs offer a significant improvement in energy density by minimizing the inactive component (*i.e.*, separators and current collectors) ratio at the device level [4]. We prepared thick electrodes (~ 80 μm) consisting of nanoscale cathode materials (NH_4_V_4_O_10_), conductive agents, and the as-synthesized S-PVdF binders in the weight ratio of 7:2:1 (Figs. 2a and S7). Next, a calendering process was carried out to increase the electrode density of the thick electrodes (Fig. 2b). Typically, most previously reported cathodes for aqueous ZIBs have been uncalendered because the calendering process decreases electrode porosity and deteriorates the pore structure of electrodes, which in turn reduces electrode wettability [27]. However, we observed that the compaction of electrodes is necessary for lowering relatively high impedance and surface roughness of the electrodes, especially for loosely packed nanoscale cathode materials with a fairly large amount of conductive agents in the thick electrodes, as shown in Fig. S8 [28]. The cathode with S-PVdF binders showed a much lower contact angle of 39° than that of the electrode with PVdF binders (97°), indicating a better surface wettability between the electrodes with S-PVdF binders and aqueous electrolytes (Fig. 2c-d). The improved wettability is beneficial for electrolyte permeability and enhanced utilization of nanoscale cathode materials in the thick electrodes.

As can be seen in Fig. 2e, many cracks were observed in the vicinity of the agglomerated active materials in the electrode with PVdF binders, which is a typical tendency when the thickness of electrodes is increased [29]. When a crack is closer to the separator, the more contributive material is excluded from the reaction, leading to an increased charge transfer resistance [30]. Meanwhile, it was difficult to find a crack on the surface of the cathode with S-PVdF binders both before and after the long-term cycling (Figs. 2f and S9c-d), resulting in smaller charge transfer resistance than that of the cathode with PVdF binders. Moreover, particle agglomeration was much more suppressed in the cathode with S-PVdF binders compared to the electrodes with PVdF binder, which is clearly seen in 3D surface roughness maps (Fig. 2g), indicating that the sulfonate groups also contributed to the dispersion of nanoscale cathode materials in the thick electrodes. To investigate the adhesive strength of S-PVdF binders-based cathodes, SAICAS experiments were conducted, as illustrated in Fig. 2h [31]. As evidenced by the amplitudes of the peaks for the S-PVdF binders versus the PVdF binders, the adhesive strength of the cathodes with S-PVdF binders was higher than the electrode prepared with PVdF binders (Fig. 2i). These observations can be attributed to the increasing polarity of S-PVdF binders due to their sulfonate groups. Therefore, this trait is useful for an electrode configuration that reduces the inactive components' ratio while maintaining battery performance.

### Electrochemical Performances of Full Cells with S-PVdF Binders

**Fig. 3:**
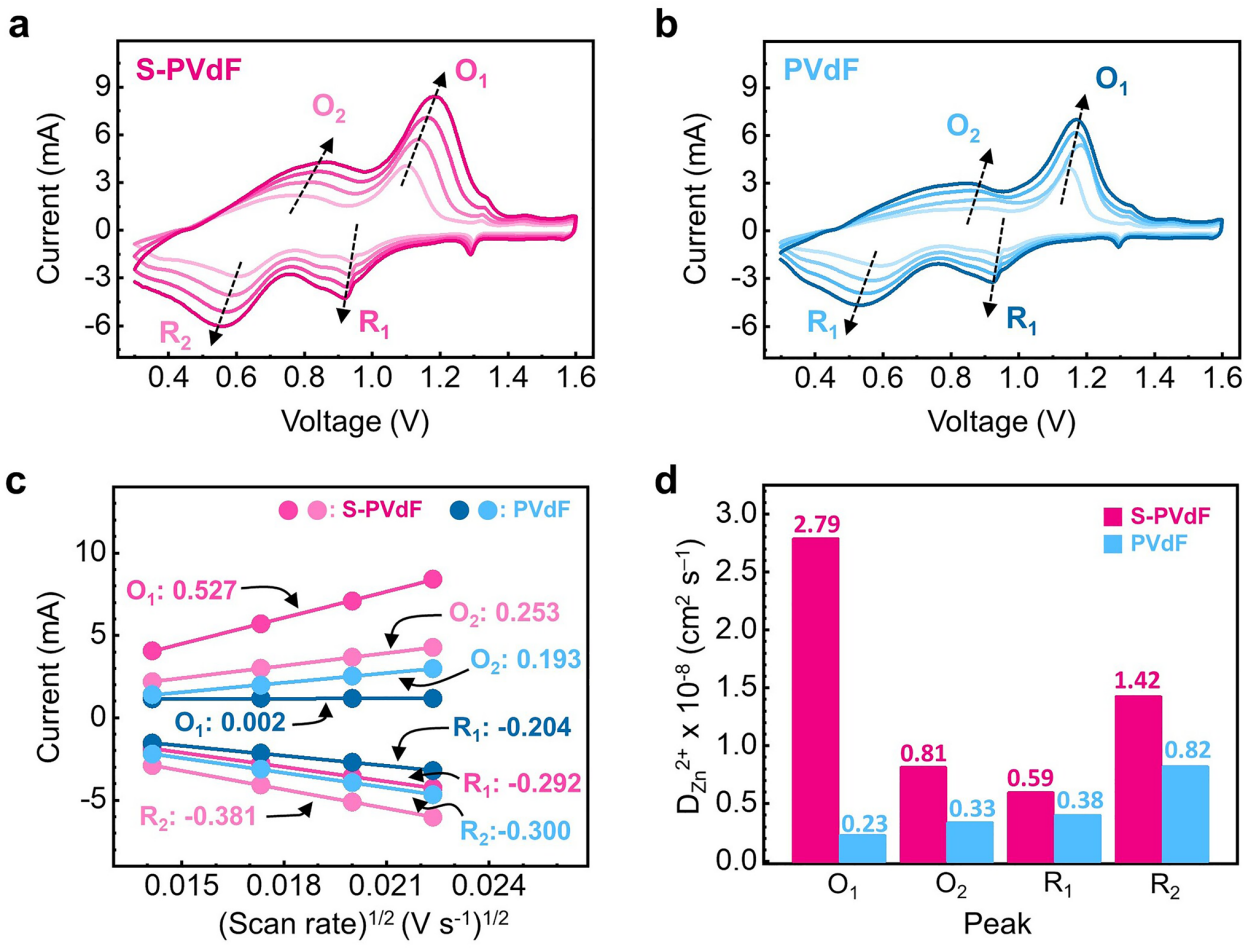
CV profiles of full cells with **a** S-PVdF and **b** PVdF binders at different scan rates (0.2 − 0.5 mV s^−1^). **c** Plots of normalized peak currents versus square root of scan rates for full cells with S-PVdF and PVdF binders S. **d** Calculated D_Zn_^2+^

To further investigate the full cell applications, electrochemical properties of the thick cathode with S-PVdF binders were evaluated by using 2032 type coin-cells coupled with zinc foil anodes, 2 M ZnSO_4_ aqueous electrolyte, and glass fiber separators. Figure S10 displays a full cell's cyclic voltammetry (CV) curves with different binders. The cell with S-PVdF binders showed a relatively smaller voltage polarization (0.17 V) between its redox potentials (1.10/0.93 V) than that of the cell with PVdF binders (0.21 V from redox potentials of 1.15 and 0.94 V) at 0.2 mV s ^−1^. These results are attributed to the high ionic conduction in S-PVdF binders due to their hydrophilicity derived from the sulfonate groups. The influence of the S-PVdF binders on the kinetic behavior of the thick electrode was also analyzed by the CV measurements at different scan rates [32]. As the scan rate increases, the amplitude of CV curves of cells with different binders increases, and distinct reduction/oxidation peaks are displayed, as can be seen in Fig. 3a, b. In addition, Zinc-ion diffusion coefficients (D_Zn_^2+^) are further calculated from the slope of the peak current and the square root of scanning rates linear fitting by the Randles–Sevcik equation [33]. As can be seen in Fig. 3c, d, the cathode with S-PVdF binders showed higher slopes than that of the electrode with PVdF binders and improved D_Zn_^2+^, revealing that facile Zn-ion conduction occurs.

**Fig. 4:**
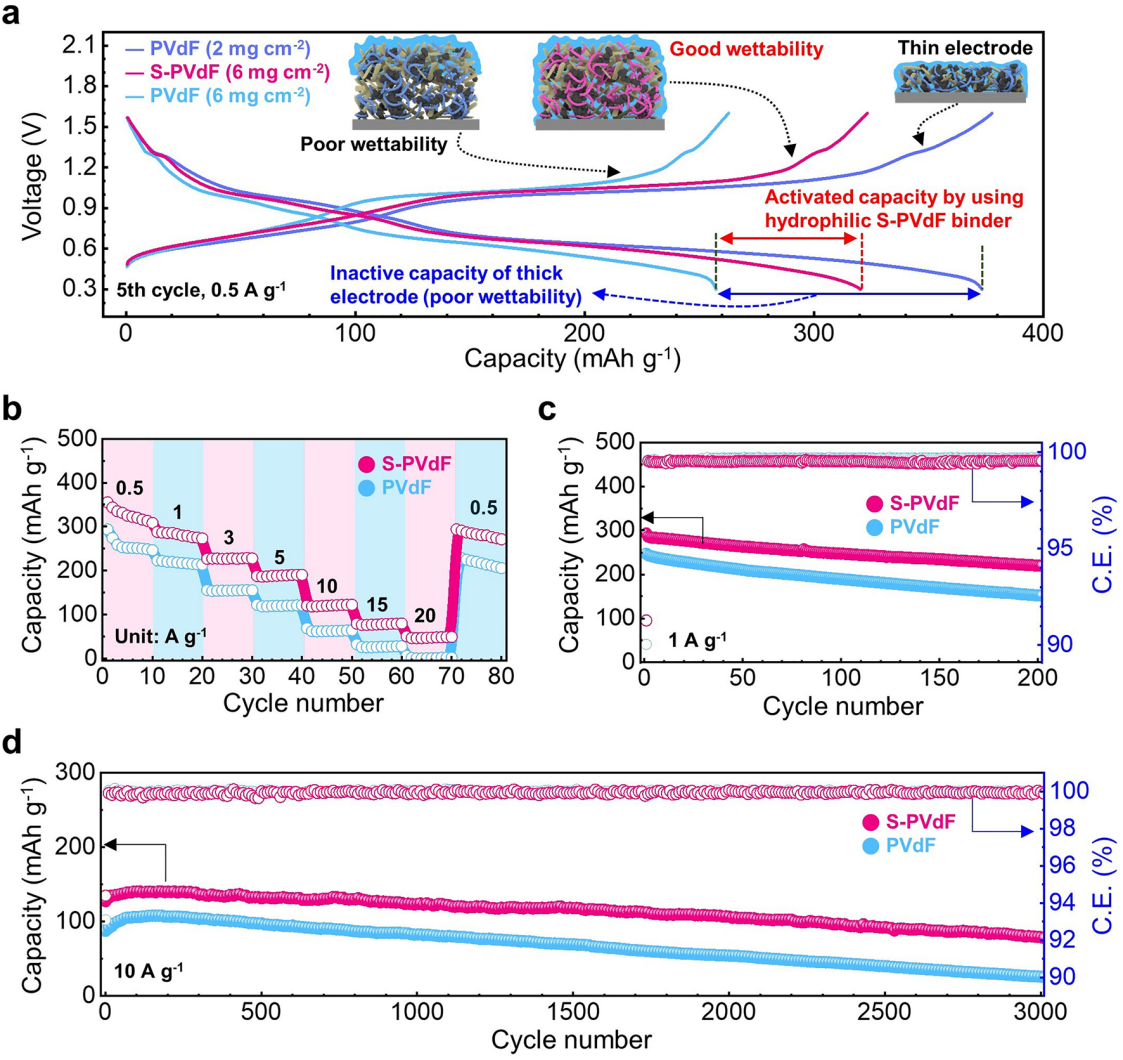
**a** Galvanostatic charge/discharge voltage profiles voltage versus specific capacity at a current density of 0.5 A g^−1^ (the 5th cycle). **b** Rate capability and **c** cycling performances at a current density of 1 A g^−1^. **d** Long-term cycling performances of full cells with different binders at a current density of 10 A g.^−1^

Figure 4a shows galvanostatic discharge/charge profiles at a current density of 0.5 A g^−1^. Although the thin electrode (~ 2 mg cm^−2^) with PVdF binders delivered high a specific capacity of ~ 373 mAh cm^−2^, much reduced capacity of ~ 257 mAh g^−1^ was obtained at the electrode with higher mass loading (~ 6 mg cm^−2^). It is noteworthy that the thick electrode with S-PVdF binders delivered a highly improved capacity of ~ 320 mAh cm^−2^ compared to the cathode with PVdF binders at the same active mass loading of ~ 6 mg cm^−2^, indicating the much better utilization of nanoscale cathode materials due to their hydrophilicity. Similarly, the cells with S-PVdF binders showed improved capacities both at rate capability and cycling tests. As shown in Fig. 4b, the cells with S-PVdF binders retain higher capacities than those with PVdF binders at all current densities, except in the thin electrode (mass loading: ~ 2 mg cm^−2^) with PVdF binders. However, the electrode with PVdF binders showed notable capacity drops at the increased mass loading of ~ 6 mg cm^−2^ (Fig. S11).

In general, electrochemical reactions in electrodes are governed by liquid-phase transport mechanisms [16]. Increasing the thickness of electrodes leads to ion transport limitations with their highly tortuous networks, which are amplified at high charge/discharge rates due to concentration gradients and subsequent electrolyte depletion [34]. Indeed, the sulfonate groups in S-PVdF binders not only improve the wettability of thick electrodes but also allow facile migration channels for large ionic transport, resulting in highly improved rate capabilities even at a very high current density of > 10 A g^−1^. The cycling performances and CE of the full cells with different binders were also measured at low and high current densities of 0.5, 1 and 10 A g^−1^, respectively (Figs. S12 and 4c, d). The full cell with S-PVdF binders showed higher reversible capacities than those with PVdF binders under both low and high current densities. In particular, the full cell with S-PVdF still maintained a high reversible capacity of ~ 78 mAh g^−1^ with a durable capacity retention of 61.2% (corresponding to an average capacity decay of 0.012% per cycle) after 3,000 cycles at 10 A g^−1^. On the other hand, only a 29.3% capacity (26 mAh g^−1^) was obtained in the full cell with PVdF binders after the long-term cycling. The capacity-increase behavior at the initial cycling segment was observed in a long-term cycling test at a high current density of 10 A g^−1^ (Fig. 4d). This phenomenon has been commonly found in nanostructured cathodes and attributed to the gradual activation of electrodes [35–40]. Interestingly, the cell with S-PVdF binders shows less capacity-increasing behavior, indicating a rapid activation process compared to PVdF binders. This is because their hydrophilicity provides sufficient wettability of aqueous electrolytes with electrodes. The galvanostatic charge/discharge voltage profiles and cycling performance of manganese-based cathode materials (δ-MnO_2_) with different binders were also evaluated. We observed a similar trend of the vanadium-based active materials (NH_4_V_4_O_10_), which indicates that the S-PVdF binders enhance the utilization of nanoscale manganese-based cathode materials in thick electrodes (Figs. S7 and S13). Furthermore, the full cell with PVdF binders showed considerable charge transfer resistance compared to the full cell with S-PVdF binders after the long-term cycles (Fig. S14).

We further evaluated the electrochemical performances of the full cells with different sulfonation degrees of S-PVdF binders (Fig. S15). The full cell with S-PVdF binders with a low *D*_s_ (5%) showed lower reversible capacities compared to the full cells with S-PVdF binders (*D*_s_: 8%) due to the insufficient electrolyte wettability (Fig. S16). Meanwhile, we were not able to prepare thick electrodes with highly sulfonated S-PVdF binders (*D*_*s*_: 15%) owing to the low viscosity of slurries. Even though the thin electrode with low mass loading of ~ 2 mg was fabricated, it showed insufficient adhesion for a fabrication process (*i.e.*, electrode punching) of cells.

**Fig. 5:**
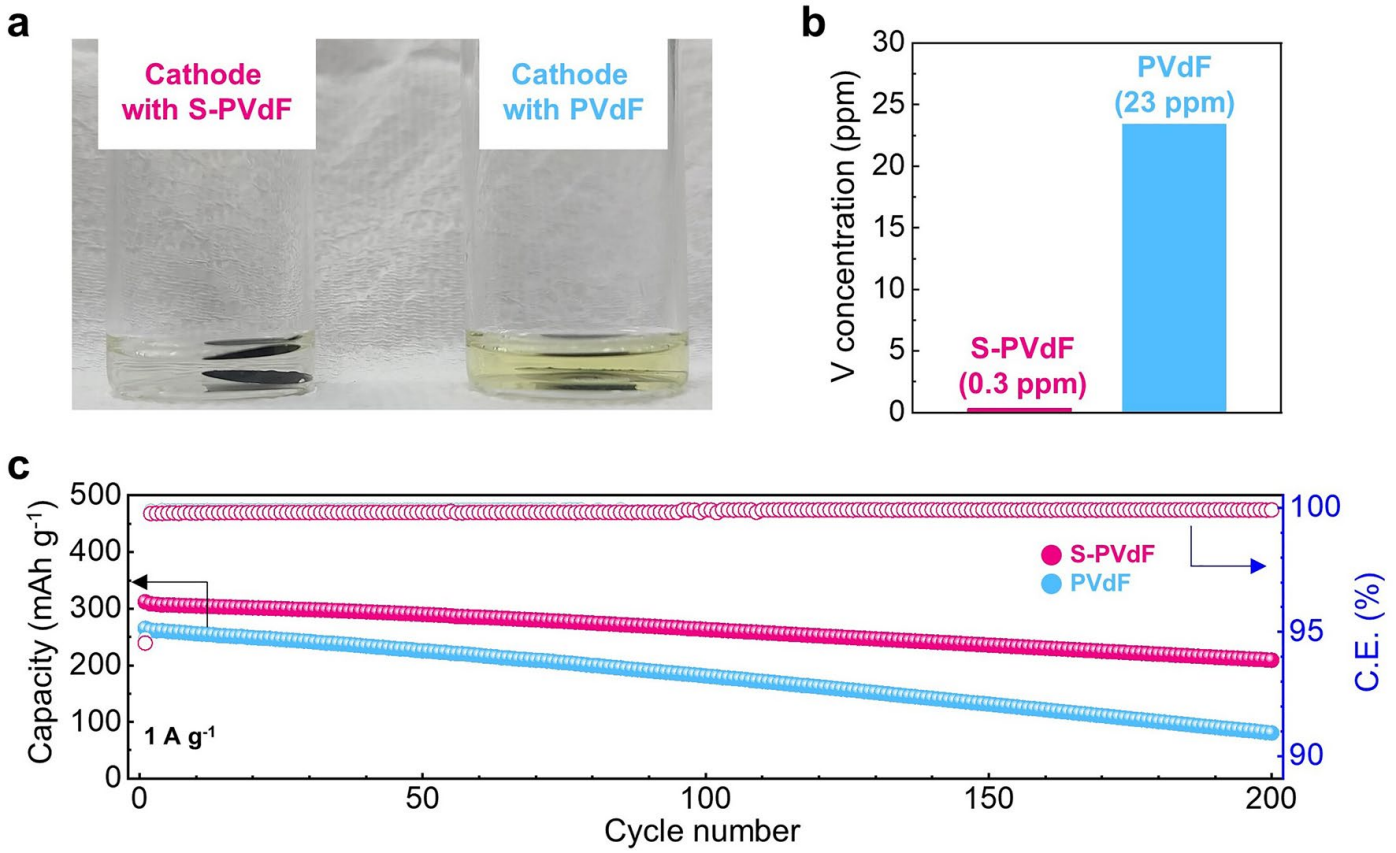
**a** Digital photograph of cathodes with different binders in 2 M ZnSO_4_ aqueous electrolyte for 72 h at 45 °C. **b** V concentration of the aqueous electrolytes with different cathodes in a. **c** cycling performances at a current density of 1 A g^−1^ at 45 °C

The cathode dissolution is one of the major issues in aqueous ZIBs, attributed to the surface vulnerability of cathode materials in aqueous electrolytes [41]. In particular, the dissolution of vanadium elements in aqueous electrolytes leads to decreased cycle stability [42, 43]. With facile ionic conduction, the S-PVdF binders also effectively restrained the dissolution of the vanadium-based cathode materials. To clarify the suppression of vanadium dissolution of vanadium-based active materials, cathodes with different binders are immersed into 2 M ZnSO_4_ aqueous electrolyte for 72 h at 45 °C (Fig. 5). The solution with S-PVdF binders remained colorless and transparent, whereas the solution with PVdF binders was light yellow, indicating vanadium dissolution from cathode materials (Fig. 5a). The inductively coupled plasma optical emission spectroscopy (ICP-OES) analysis exhibits that the dissolved vanadium (V) amount of the electrode with PVdF binders is almost 77 times higher than that of the electrode with S-PVdF binders (Fig. 5b). As shown in Fig. S17, X-ray photoelectron spectroscopy (XPS) results confirmed the absorbed V species (VO) on the S-PVdF films. In mild acidic solutions, sulfonate groups usually have a high affinity to heavy metal ions and can chelate them [44]. Therefore, the sulfonated PVdF binders effectively suppressed cathode dissolution, resulting in enhanced capacity retention at higher temperature operations. This V chelating ability of S-PVdF binders is especially beneficial for cycling at elevated temperatures. The full cell with PVdF binders showed poor cycle stability during the cycle test at 45 °C. In contrast, the full cell with S-PVdF binders delivers much higher reversible capacities with highly improved cycle stability at an elevated temperature, as seen in Fig. 5c. Such a remarkable improvement in the cycle performance demonstrates that the S-PVdF binders effectively prevents the cathode dissolution of vanadium-based active materials.

## Conclusions

In summary, hydrophilic binders for aqueous ZIBs were synthesized by a simple sulfonation process of PVdF, considering physicochemical properties such as adhesion and processability. Abundant sulfonate groups in S-PVdF binders allowed fast and sufficient electrolyte wetting as well as improved ionic conduction in thick electrodes, enabling enhanced utilization of nanoscale cathode materials in the thick electrodes with high mass loading of ~ 6 mg cm^−2^. As a result, the full cell with S-PVdF binders showed highly improved reversible capacities under a high current density of 10 A g^−1^ and, especially, a durable capacity retention of 61.2% (corresponding to an average capacity decay of 0.012% per cycle) after 3,000 cycles at 10 A g^−1^ compared to the full cell with PVdF binders. Moreover, the full cell with S-PVdF binders delivers much higher reversible capacities with highly improved cycle stability at elevated temperatures due to the V chelating ability of S-PVdF binders in aqueous electrolytes. Our strategy will provide insightful and feasible guidelines for developing high-energy-density aqueous ZIBs.

### Supplementary Information

Below is the link to the electronic supplementary material.

Supplementary file1 (DOCX 2413 KB)
