# Supplementary material for: Enhancing Hydrophilicity of Thick Electrodes for High Energy Density Aqueous Batteries
Source: Nanomicro Lett. 2023 Apr 10;15:97. doi: 10.1007/s40820-023-01072-y (PMC10086092; doi:10.1007/s40820-023-01072-y)
Supplement: Supplementary file 1 — Supplementary file1 (DOCX 2413 KB) [file 40820_2023_1072_MOESM1_ESM.docx]

Supporting Information

**Enhancing Hydrophilicity of Thick Electrodes for High Energy Density Aqueous Batteries**

Jungeun Lee^1^, Hyeonsoo Lee^1^, Cheol Bak^2^, Youngsun Hong^1^, Daeha Joung^3^, Jeong Beom Ko^1^, Yong Min Lee^2,^* and Chanhoon Kim^1,^*

^1^Sustainable Technology and Wellness R&D Group, Korea Institute of Industrial Technology (KITECH), 102 Jejudaehak-ro, Jeju-si, Jeju-do 63243, Republic of Korea

^2^Department of Energy Science and Engineering, Daegu Gyeongbuk Institute of Science and Technology (DGIST), Daegu 42988, Republic of Korea

^3^Department of Physics, Virginia Commonwealth University, Richmond, VA 23284, USA

*E-mail: ckim8608@kitech.re.kr

**Keywords**: thick electrodes, hydrophilic binder, sulfonation, aqueous zinc-ion batteries, high areal capacity

**
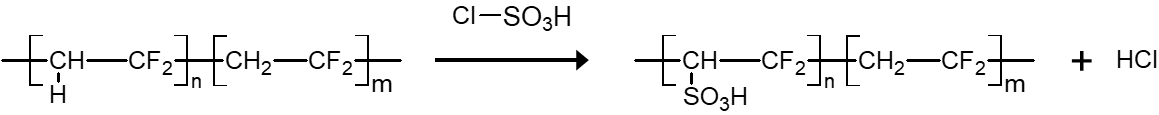
**

**Fig. S1** A scheme to synthesize sulfornated PVdF (S-PVdF) from PVdF through simple chemical reaction.

**
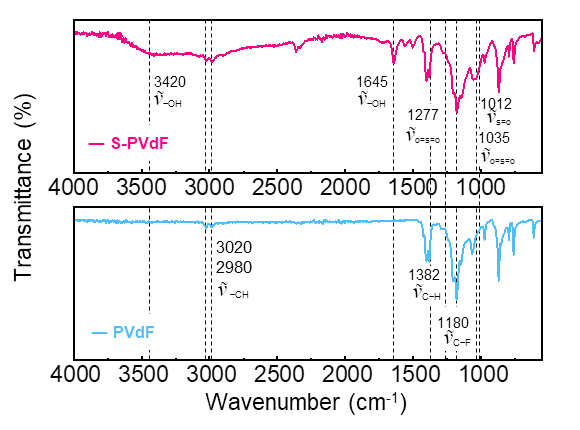
**

**Fig. S2** FT-IR spectra of S-PVdF and PVdF binders.


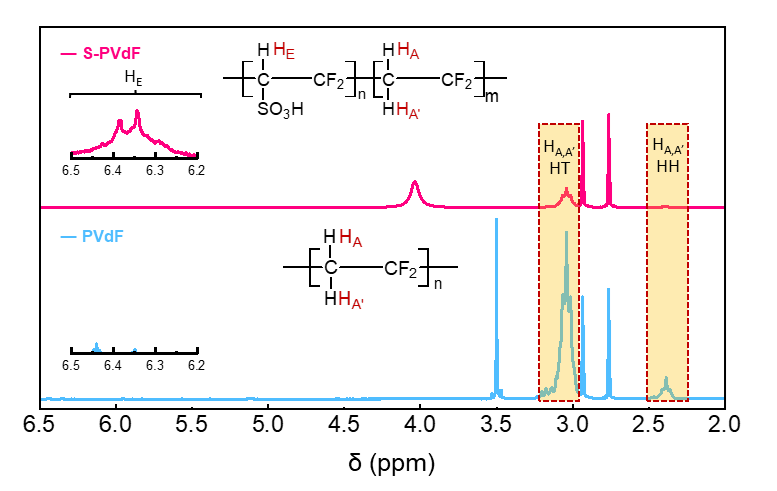


**Fig. S3** ^1^H NMR spectra of S-PVdF and PVdF binders.


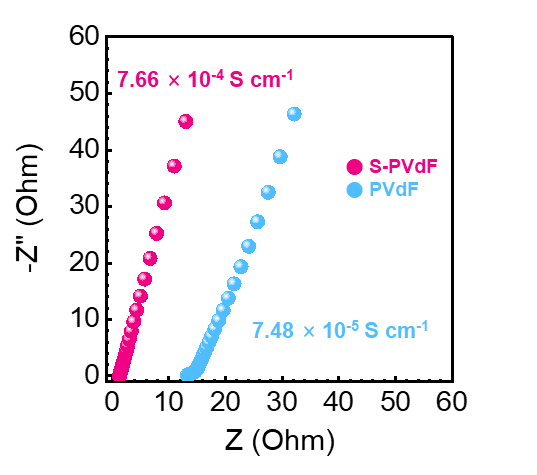


**Fig. S4** Nyquist plots of S-PVdF and PVdF binders and their ionic conductivities.


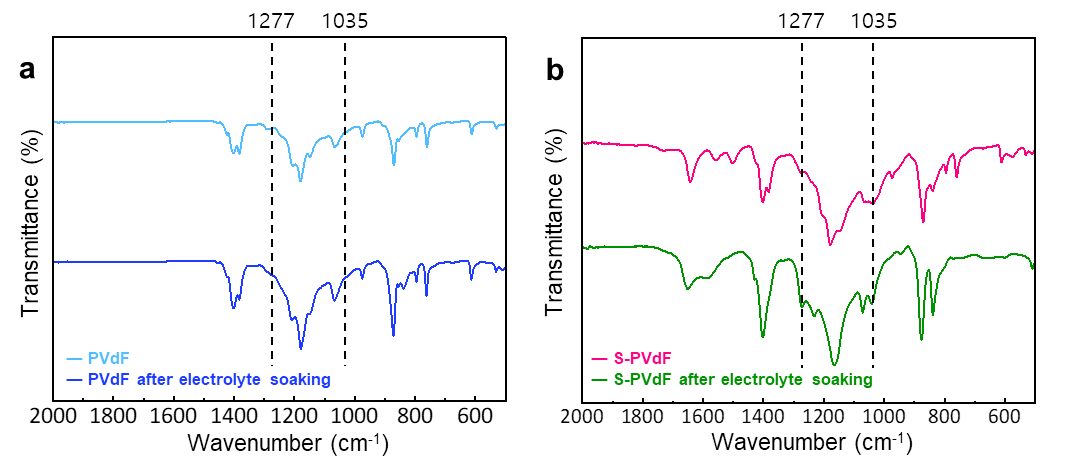


**Fig. S5** FTIR spectra of binder before and after electrolyte soaking. **a** PVdF binders before and after electrolyte soaking. **b** S-PVdF binders before and after electrolyte soaking.

We have confirmed that the sulfonate groups in S-PVdF binders can provide abundant coordination sites with Zn^2+^ by Fourier transformation infrared spectrum (FTIR). After soaking in electrolyte for 24 h, the 1277 cm^−1^ symmetric O=S=O peak shifts to a wave number (1271 cm^−1^) in S-PVdF binders, which suggests that the Zn^2+^ ions interact with sulfonate groups [1].


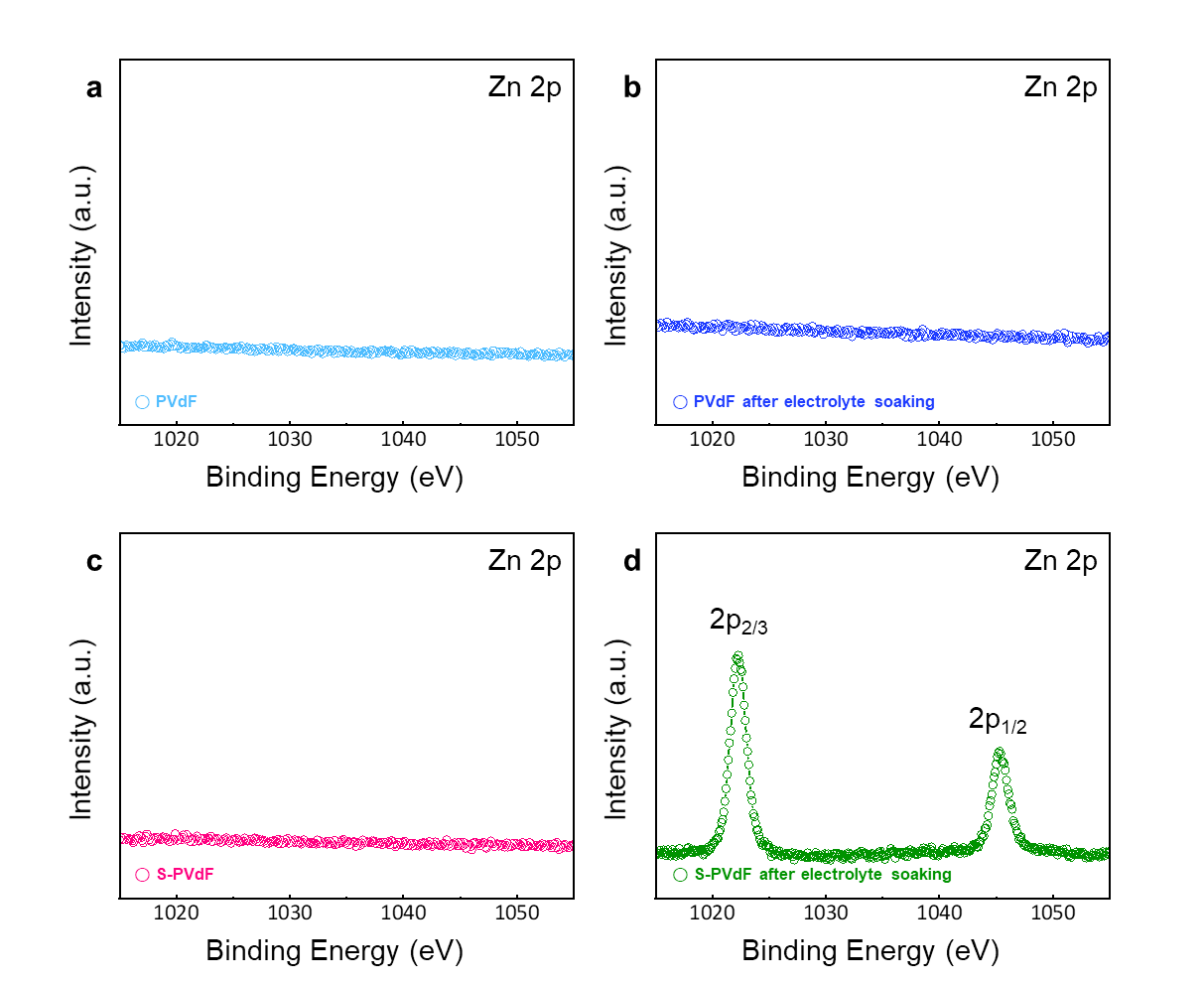


**Fig. S6** XPS spectrum of Zn 2p in PVdF binders **a** before and **b** after electrolyte soaking. XPS spectrum of Zn 2p in S-PVdF binders **c** before and **d** after electrolyte soaking.

We also measured the binding energy of PVDF and S-PVDF to zinc ions via X-ray photoelectron spectroscopy (XPS). As can be seen in Fig. S6, XPS analysis exhibits the characteristic binding energies of Zn 2p_3/2_ and Zn 2p_1/2_ of 1022.3 and 1045.4 eV in S-PVdF [2].


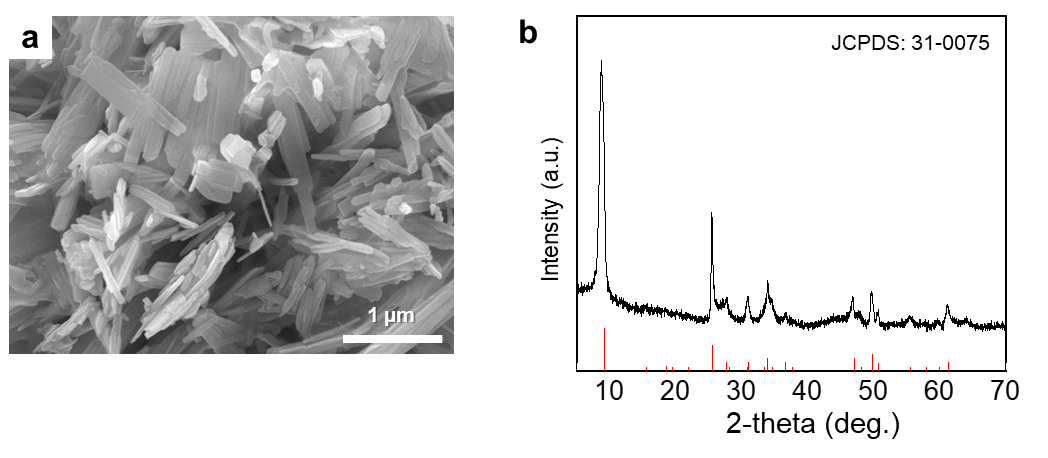


**Fig. S7 a** SEM image and **b** XRD pattern of NH_4_V_4_O_10_ (NHVO).


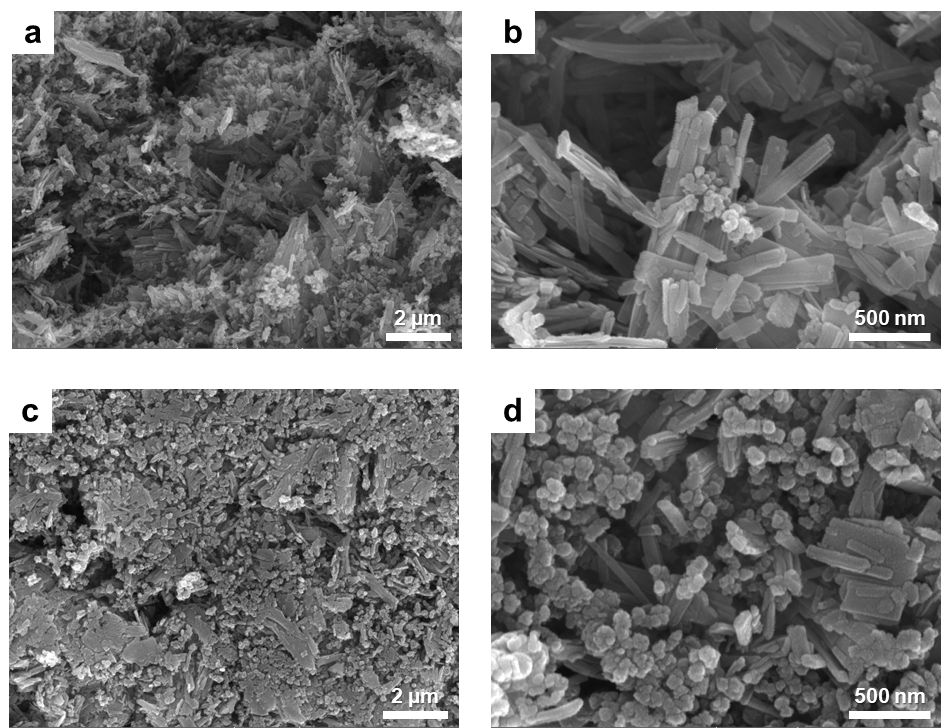


**Fig. S8** Cross-sectional SEM image of a cathode with **a** S-PVdF binders and **b** its magnification. Cross-sectional SEM image of a cathode with **c** S-PVdF binders and **d** its magnification after the calendering process.

**
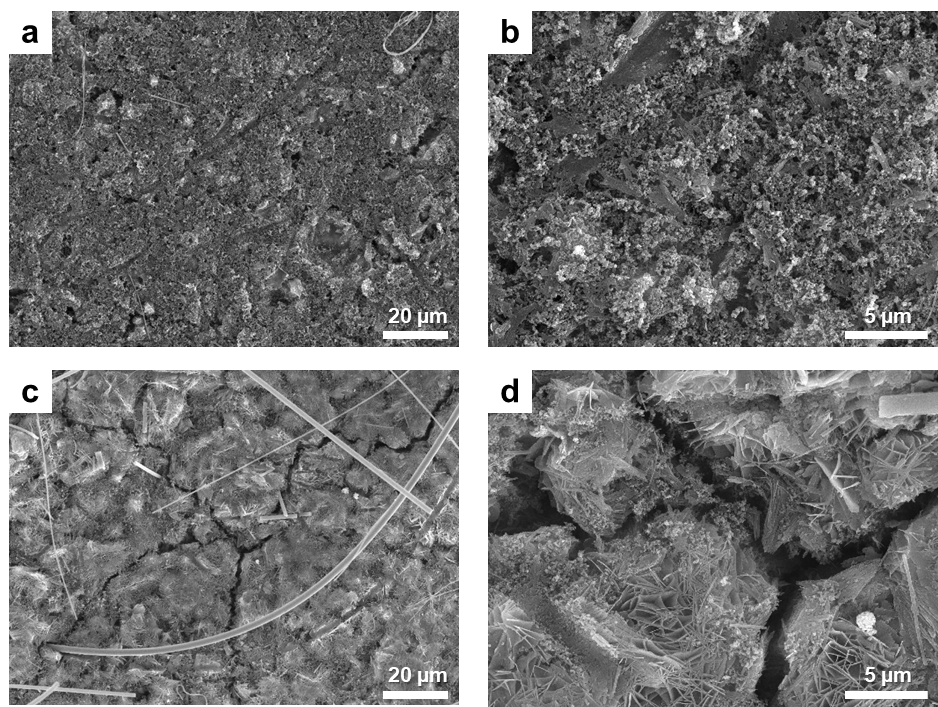
**

**Fig. S9** **a** SEM images of cathodes with S-PVdF binders and **b** its magnification. SEM images of cathodes with **c** PVdF binders and **d** its magnification.


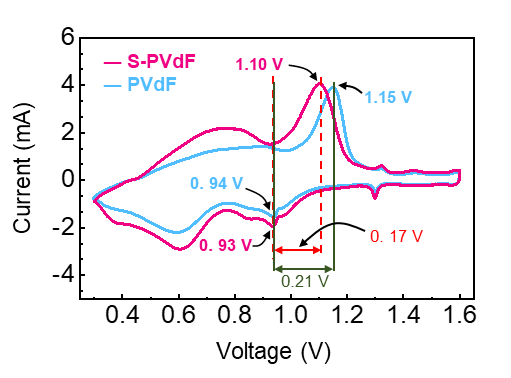


**Fig. S10** CV profiles of full cells with S-PVdF and PVdF binders at 0.2 mV s^−1^.


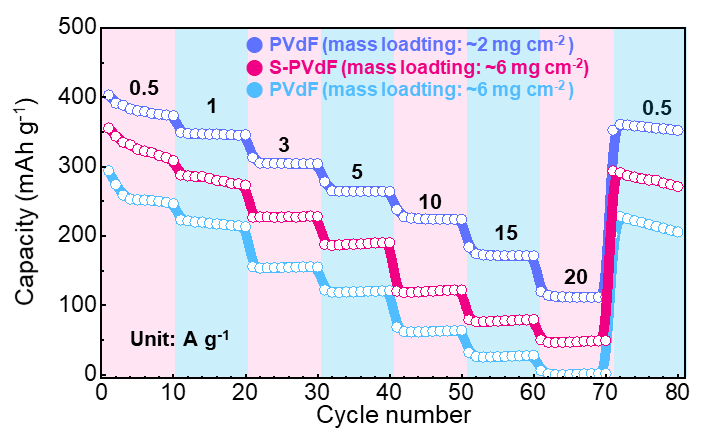


**Fig. S11** Rate capability of cathodes with different binders.


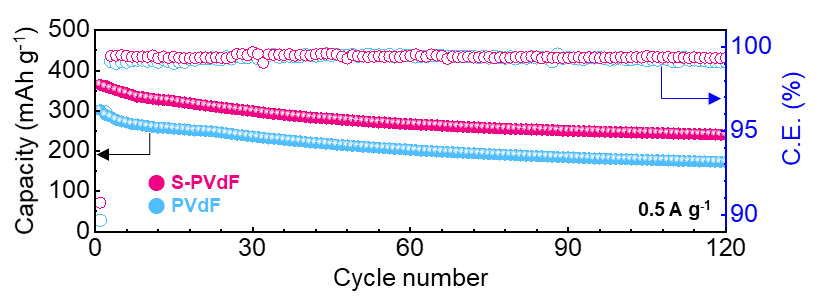


**Fig. S12** Cycling performances of full cells with different binders at a low current density of 0.5 A g^-1^ (~0.1 C).

**
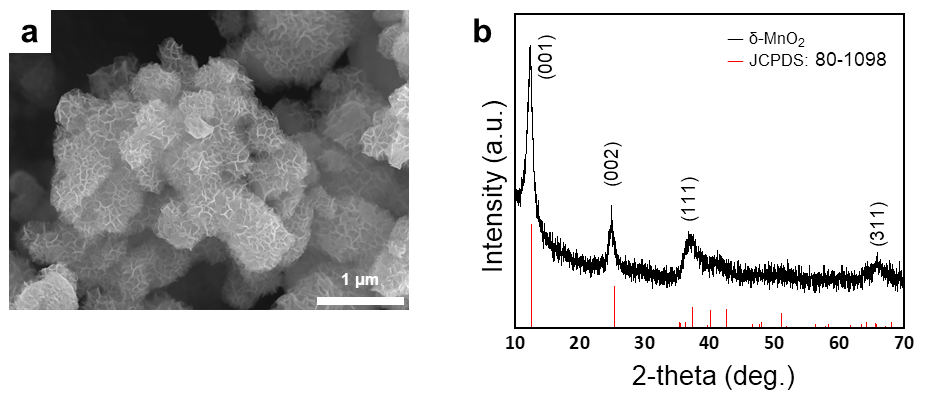
**

**Fig. S13** **a** SEM image and **b** XRD pattern of δ-MnO_2_.

To prove the universality of the binder, δ-MnO_2_ was prepared. First, 1.2g of KMnO_4_ and 0.215 g of MnSO_4_ were dissolved in 130 ml DIW. Then, the solution was put into a Teflon-contained autoclave and heated at 160 °C for 12 h. After cooling and centrifugation, the product was rinsed with DIW several times. Finally, δ-MnO_2_ was obtained after drying in a convection oven at 80 °C for 12 h. Next, the δ-MnO_2_ was cast on stainless (SUS) foil and composed of three components: active material, conductive materials, and binders in the weight ratio of 7:2:1. Denka black was selected as the conductive material. Slurries containing the three components in N-methyl-2-pyrrolidone (NMP) were cast onto SUS foil (20 µm thick) via the doctor blading method, followed by a drying step at 80 °C for 12 h under vacuum. The active mass loadings for the cathode materials were ~ 3 mg cm^-2^. The full cell with S-PVdF binders showed a higher reversible capacity (282 mAh g^−1^) than those with PVdF binders (237 mAh g^−1^) at the same mass loading of 3 mg cm^−2^) at a current density of 0.2 A g^−1^. Moreover, the full cell with S-PVdF showed higher reversible capacities in the cycle test. More importantly, it retained a highly improved capacity of ~ 185 mAh g^−1^ with a durable capacity retention of 66% after 200 cycles at 0.2 A g^−1^. On the other hand, only a 20% capacity (49 mAh g^−1^) was obtained in the full cell with PVdF binders after the cycle test.


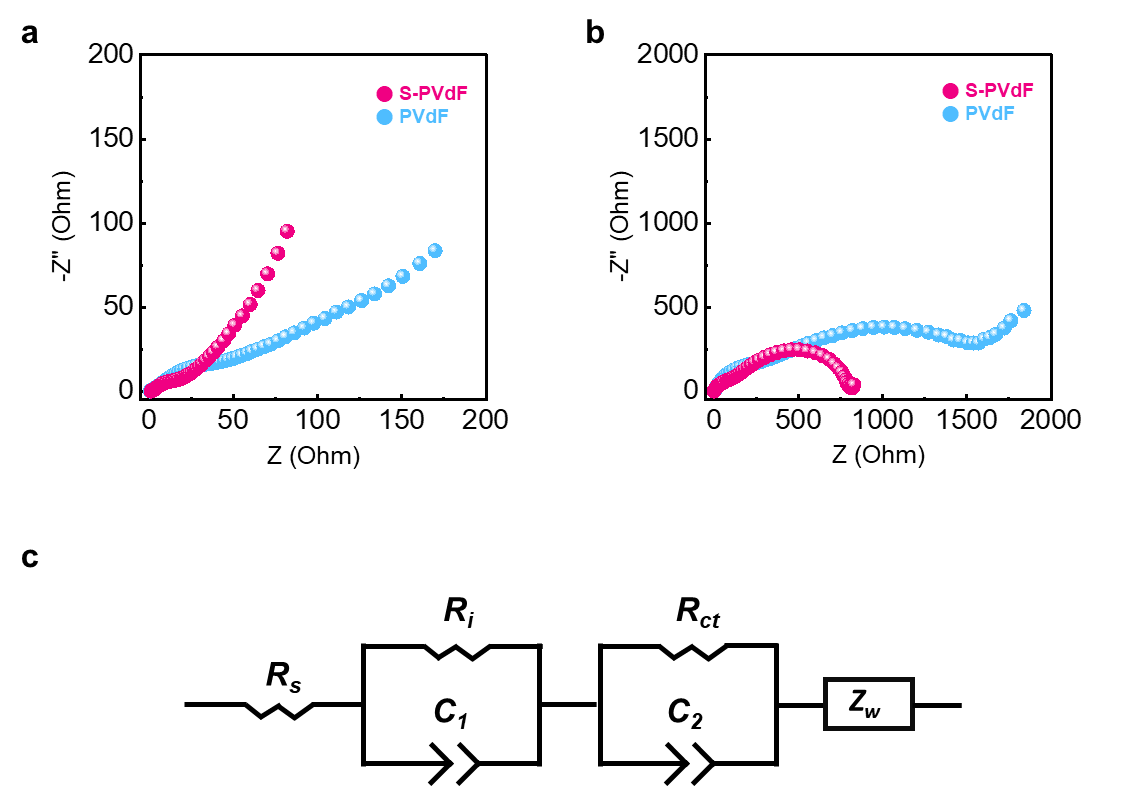


**Fig. S14** EIS spectra of **a** before and **b** after long-term cycling (3000 cycles at 10 A g^−1^). **c** Corresponding equivalent circuit. R_i_ and R_s_ are the ohmic resistance of solution and electrodes. R_ct_ is the charge-transfer resistance. C_1_ and C_2_ are the constant phase element of the double-layer capacitance, respectively. Zw is the Warburg impedance.

**
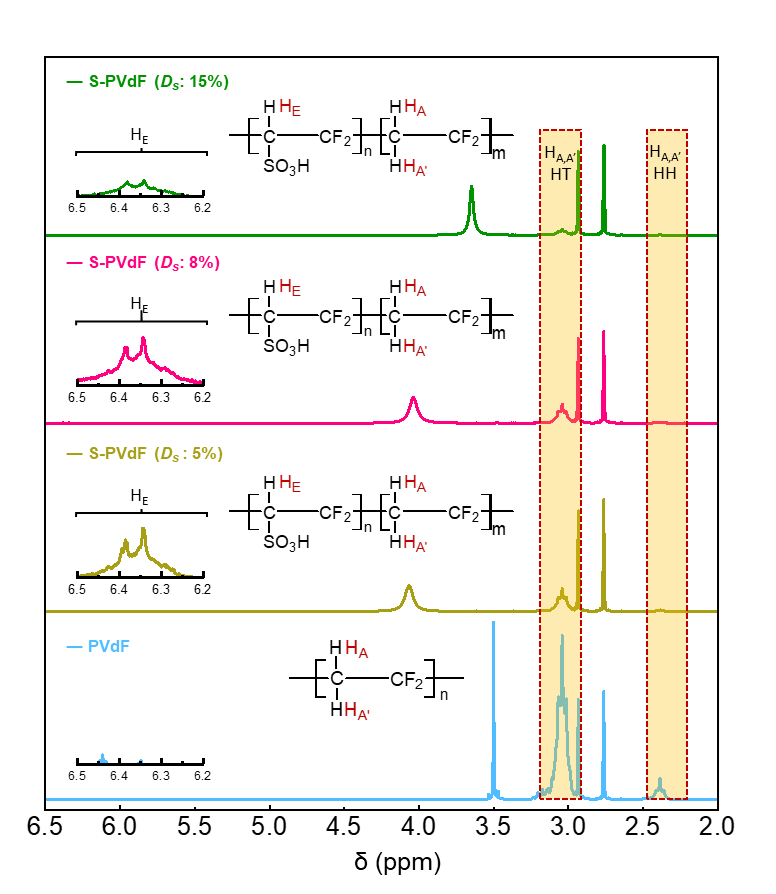
**

**Fig. S15** ^1^H NMR spectra of binders with different sulfonation degrees.

**
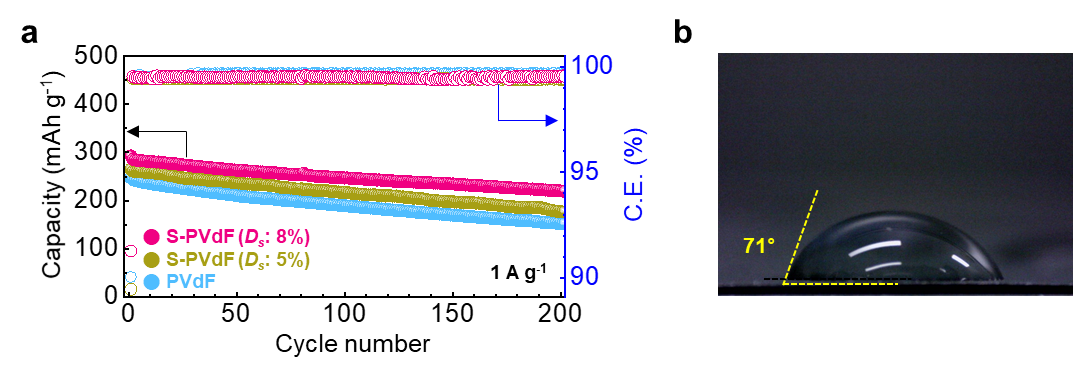
**

**Fig. S16** **a** Galvanostatic cycling performances of cathodes with different binders at a current density of 1 A g^-1^. **b** Contact angle of the cathode with S-PVdF binder (low sulfonation degree of 5%).


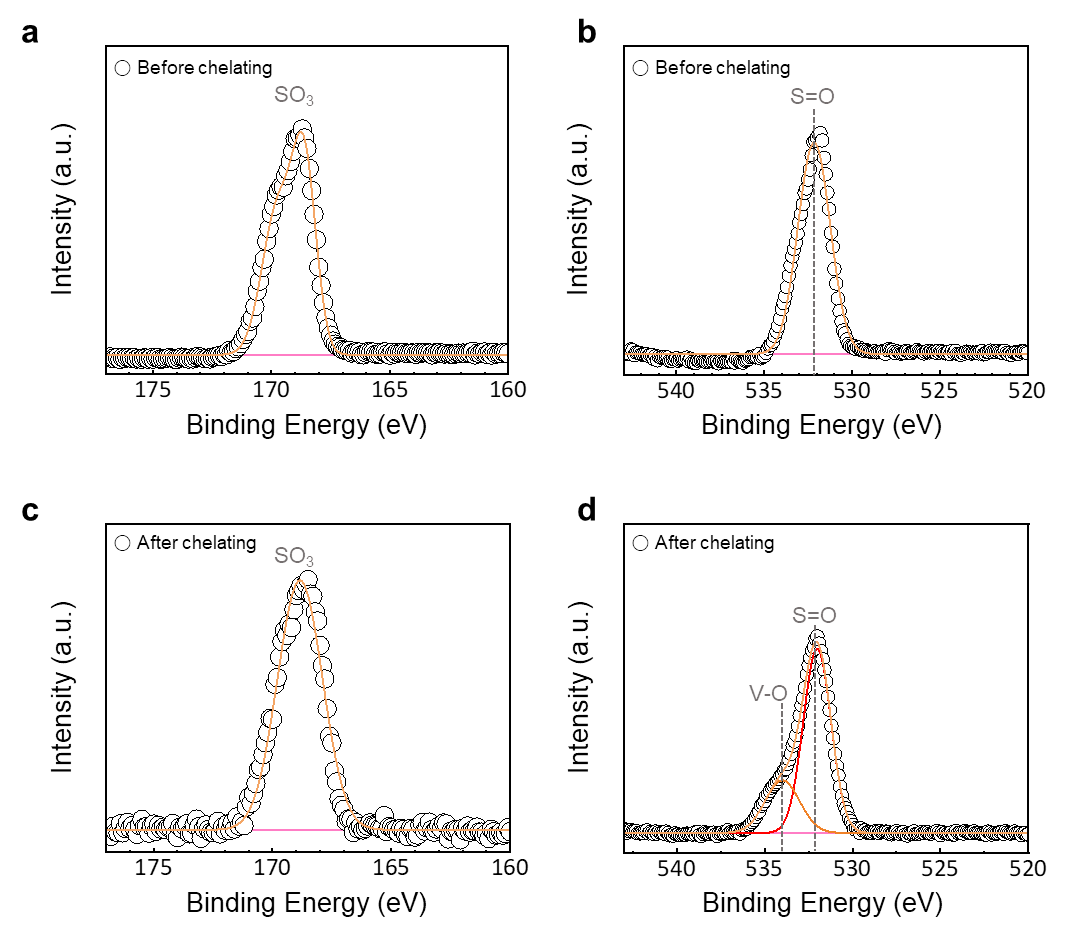


**Fig. S17** High-resolution XPS spectra of S-PVdF films before and after being immersed into cathode-dissolved electrolytes. **a** S2p and **b** O1s spectra peaks of immersed S-PVdF film before being immersed into cathode-dissolved electrolytes. **c** S2p and **d** O1s spectra peaks S-PVdF film after being into cathode-dissolved electrolytes.

To clarify the suppression of vanadium dissolution of vanadium-based active materials, we prepared cathode-dissolved electrolyte. First, 0.1 g of the vanadium-based active materials (NH_4_V_4_O_10_) were immersed into 10 ml of 2 M ZnSO_4_ aqueous electrolytes. Then, the solution was kept in an oven for 24 h at 70 °C and puriﬁed via ﬁltration by 0.45 µm syringe ﬁlter. S-PVdF films for measuring ionic conductivities were also used here. The films were immersed into the yellowish cathode-dissolved electrolyte for 24 h at room temperature. Then, the films were rinsed with DIW and ethanol several times. Finally, the films were dried in a convection oven at 80 °C for 12 h.

**Supplementary References**

[S1] H. Fan, M. Wang, Y. Yin, Q. Liu, B. Tang, et al., Tailoring interfacial Zn^2+^ coordination via a robust cation conductive film enables high performance zinc metal battery. Energy Storage Materials. **49**(380-389 (2022).
https://doi.org/https://doi.org/10.1016/j.ensm.2022.04.031

[S2] Y. Yang, C. Liu, Z. Lv, H. Yang, Y. Zhang, et al., Synergistic manipulation of Zn2+ ion flux and desolvation effect enabled by anodic growth of a 3d ZnF2 matrix for long-lifespan and dendrite-free zn metal anodes. Advanced Materials. **33**(11), 2007388 (2021).
https://doi.org/https://doi.org/10.1002/adma.202007388
